# Pessimistic health and optimistic wealth distributions perceptions in Germany and the UK: evidence from an online-survey

**DOI:** 10.1186/s12889-021-11355-x

**Published:** 2021-07-03

**Authors:** Luka J. Debbeler, Harald T. Schupp, Britta Renner

**Affiliations:** 1 Psychological Assessment and Health Psychology, Department of Psychology, University of Konstanz, P.O. Box 47, D-78457 Constance, Germany; 2 General and Biological Psychology, Department of Psychology, University of Konstanz, P.O. Box 36, D-78457 Constance, Germany

**Keywords:** Health, Inequality, Misperception, Public health, Fairness, Wealth

## Abstract

**Background:**

Inequalities in health and wealth distributions are becoming pressing societal problems in many countries. How these inequalities are perceived and to what degree perceptions are aligned with actual distributions, is important for trust in public health services, social and economic policies, and policymakers. This study aims to assess perceived and desired levels of inequality in health and wealth in Germany and the UK.

**Methods:**

The online-survey was filled out by 769 volunteers (322 from Germany, 447 from the UK), recruited from an existing commercial panel (Prolific Academic) or via Facebook advertisements in 2019. Perceived and ideal national health and wealth distributions were assessed and compared to actual health indicators (i.e. days absent from work, number of visits to general practitioners (GPs) and self-rated health), and actual wealth distributions with t-tests.

**Results:**

A pronounced gap emerged between the estimated, ideal and actual inequality. Both samples strikingly underestimated the proportion of (very) good health in the national distribution by a factor of ~ 2.3 (participants estimated that 34% of the German and 36% of the UK population respectively are very healthy or healthy, while the actual proportion in the population was 75% in Germany and 84% in the UK, *P* < 0.001 for all). Moreover, actual health distributions were much closer to the desired than the perceived health distributions (78% of German and 72% of UK participants ideally being very healthy or healthy). A reversed pattern of results emerged for wealth in both samples, with wealth inequality being strikingly worse than desired and inequality being underestimated by a factor ~ 1.7 (*P* < 0.001 for all). Results were consistent across demographic groups.

**Conclusions:**

Respondents in both Germany and the UK have profoundly negative misperceptions regarding the distribution of health, which contrasts with starkly positive misperceptions regarding the distribution of wealth, indicating that the public is healthier but poorer than they think. More importantly, from a public health perspective, a high level of consensus emerged, with both healthy and wealthy participants misperceiving health and wealth distributions.

**Supplementary Information:**

The online version contains supplementary material available at 10.1186/s12889-021-11355-x.

## Introduction

Health and wealth inequalities are observed in all societies. Although some inequalities may be considered unavoidable, resulting from sociodemographic characteristics such as age and gender, many of these inequalities or disparities are potentially amenable to public health and policy interventions [1, 2]. For health, many countries have amended the goal of universal health care proposed by the WHO [3, 4] but healthcare systems are challenged by pandemics such as the coronavirus disease (COVID-19) outbreak, increasingly elderly societies [5], and risky lifestyle habits [6], which are fueling heated debates about public spending on healthcare [7]. Likewise, increasing wealth inequality is becoming a pressing societal problem in many countries [8]. Despite widespread agreement that an ideal society would provide good health and a modest wealth gap, determining the ideal societal distribution of health and wealth is a complex question and a source of friction between stakeholders. Which distribution of health and wealth in a society are deemed just and to what degree they are aligned with actual distributions, is important for trust in public health services, social and economic policies, and policymakers [9–11]. Previous research showed that respondents from countries with relatively high levels of wealth inequality (e.g., the United States (US) and Australia) dramatically underestimated the actual level of wealth inequality [11–13]. Moreover, they constructed an ideal wealth distribution that was far more equitable than even their low, misperceived estimates of the actual wealth distribution. Specifically, respondents from the US and Australia estimated that the richest 20% of the population owned around 59 and 47% of total wealth, when the actual numbers were 84 and 62%, but believed that this richest quintile should ideally own slightly under 34% [11, 13]. Hence, respondents showed profoundly positive misperceptions of how wealth was distributed, and a desire for a much more modest wealth gap. While inequalities in wealth were markedly underestimated, they were not generally seen as unjust, which raises the question of whether a similar pattern emerges for health. Since health is a vital resource people might wish that, in contrast to wealth, it should be accessible for the vast majority of society. Thus, more vulnerable groups might desire greater health along with the healthy and wealthy, resulting in a shared consensus that the ideal distribution of health is good health for everybody.

We chose to sample citizens from Germany and the United Kingdom (UK), since there are several key similarities as well as differences between the two countries’ health care systems. Although life expectancy in Germany is no higher than in the UK, the German population is ageing more rapidly. Germany’s spending on health care is relatively high, at just over 11.3% of its wealth, compared to 9.6% in the UK [14]. It has more doctors and hospital beds per patient than the UK [15]. Nine out of 10 Germans pay 7% of their pre-tax salary into statutory health insurance, which is matched by their employers [16, 17]. Throughout the UK, the NHS is paid for out of general taxation and the proportion of public money allocated to it is a matter of policy for each of the devolved governments.

The purpose of the current study was to assess whether respondents from Germany and the UK misperceive their countries’ health distributions, and wish for their societies to be more healthy. We hypothesized that there is a consensus across both samples and demographic groups regarding perceived and ideal health distributions, and that these would also be related to objective indicators of the nation’s health, which would in turn inform whether health care efforts are perceived accurately in relation to national health. Overall, the study addresses the following four research questions: 1) How is the actual distribution of health in Germany and the UK perceived, and the ideal distribution of health construed? 2) What is the relationship between the perceived, ideal, and actual national distributions of health? 3) Do estimates differ between respondents from Germany and the UK pointing towards the impact of differences in health systems, or are they similar, providing first evidence for a more general finding on the perception of health distributions? Likewise, do estimates differ between demographic groups? 4) Are there systematic differences across the domains of health and wealth?

## Methods

### Procedure

The German questionnaire was pre-tested in a pilot study and then adapted before data collection. The English version was created and then back translated by native and bilingual English and German speakers (see supplementary material for the English questionnaire). Qualtrics (SAP SE., 2018) was used to conduct online questionnaires in Germany between January 11th and February 17th 2019, and in the UK between July 22nd and August 8th 2019. The first page of the questionnaire contained a brief introduction. Participants gave informed consent by indicating that they had read and understood the study information and wanted to participate. It took about 10–15 min to complete the questionnaire, and participation was entirely voluntary. The study was conducted in accordance with the ethical guidelines of the German Psychological Association (DGPs). The institutional review board (IRB) of the University of Konstanz classified the study as non/minimally invasive (34/2018). Data for the objective national health distributions were drawn from the European Health Interview Survey (EHIS) [18] provided by Eurostat (project number RPP 260/2019-EHIS), which is compulsory for all European member states based on the European Commission Regulation 141/2013. EHIS assesses standardised health indicators every 5 years to allow comparisons within and between European countries. The present study includes data collected during the second EHIS wave (2015 in Germany, 2013 in the UK). Data for objective national wealth distributions were drawn from a report on wealth distribution from the German Institute for Economic Research (40/2019) [19] and the data set “Total Wealth: Wealth in Great Britain” of the National Office for Statistics [20].

#### German survey sample

A total of 417 participants were recruited through postings and social media advertisements on Facebook. Of these, 90 were excluded from the analyses due to missing data and five because they did not fulfill the study requirements (missing study consent; not resident in Germany). A final sample of 322 participants was included in the analysis (74% female). The participants had a mean age of 31 years (range 18–68, *SD* = 11.9) and a mean BMI of 22.9 (range 16–42, *SD* = 3.38). For compensation, participants could take part in a raffle for 50€ Amazon vouchers.

#### United Kingdom survey sample

The participants (*n* = 443) were recruited via the online research platform Prolific Academic (https://www.prolific.co/) and through postings and social media advertisements on Facebook (*n* = 52). Forty-eight of the recorded datasets were excluded (*n* = 20 duplicates due to technical reasons; *n* = 19 due to missing data, *n* = 9 because they did not fulfill the study requirements (not being of age; not resident in the UK)). A final sample of 447 participants was included in the analysis (71% female). The participants had a mean age of 35 years (range 18–78, *SD* = 12.0) and a mean BMI of 26.5 (range 11–50, SD = 6.1). For compensation, participants recruited on Facebook could take part in a raffle for £50 Amazon vouchers. The reimbursement of participants from Prolific Academic was based on the platform’s recommendations (https://www.prolific.co/).

### Materials

#### Demographics

Participants were asked to provide their gender, year of birth, country of residence, highest educational level (school certificate or exam qualification), highest graduation level (degree), and their height and body weight.

#### Perceptions of actual and ideal national health distributions

Based on the procedure suggested by Norton and Ariely [11, 21], respondents were asked to estimate the actual distribution of health within their own country’s population by the item “How healthy is the [German/ UK] population?”, and what they would see as the ideal distribution by the item “How healthy should the [German/ UK] population ideally be?”. The participants were further asked to think of five different health categories ranging from *very healthy* [1] to *very unhealthy* [5] and to indicate what percentage of their countries’ population belonged in each category. These percentages were then set on a bar chart scaled from 0 to 100%. The sum of set bar percentages could not exceed 100%. An example that included a visual representation was provided as an illustration.

#### Perceptions of actual and ideal national wealth distributions

The participants’ perceptions of actual national wealth distributions were assessed by the item “How is wealth distributed in [Germany/the UK]?”. Respondents indicated the percentage of their countries’ total wealth that they thought was owned by each wealth quintile (from the richest 20% to the poorest 20%) [11]. They were also asked to indicate how total wealth should ideally be distributed across the wealth quintiles. Percentages for each wealth quintile could be set on a bar chart scaled from 0 to 100%. The sum of set bar percentages could not exceed 100%. Following Norton and Ariely [11], we defined wealth as the sum of all major assets owned in a household (e.g. house, car, cash, saving, shares, etc.) minus any debts (e.g. car loans, mortgage, loans, etc.).

#### Self-rated health and wealth

Self-rated health was assessed in five categories from *very healthy* [1], *healthy* [2], *fairly healthy* [3], *unhealthy* [4], to *very unhealthy* [5]. Self-rated wealth was assessed within the five categories richest *20%* [1], *second 20%* [2], *middle 20%* [3], *fourth 20%* [4] and *poorest 20% of the population* [5].

The online survey included additional items which assessed smoking status, risk perception, and variables regarding physical fitness, body weight and life-expectancy, with some variables serving to replicate standard findings on optimistic bias and others that were not relevant to the present paper.

#### Actual national health distribution in Germany and the United Kingdom

National representative data on health indictors were drawn from EHIS data sets for Germany and the UK (EHIS, project number RPP 260/2019-EHIS) [18]. According to previous studies, the number of days absent from work due to personal health problems and the number of doctor visits were used as objective health indicators, which covary also with subjective health from very health to very unhealthy (e.g., [22, 23]). Data were categorized into five health categories (*very healthy* [1] to *very unhealthy* [5]), including the number of days absent from work due to personal health problems in the past 12 months *0 days* [1], *1–7 days* [2], *8–14 days* [3], *15–30 days* [4] and *> 30 days* [5], the number of times they had consulted a general practitioner or family doctor during the past 4 weeks for personal treatment with *0 times* [1], *1 time* [2], *2 times* [3], *3–4 times* [4], *> 4 times* [5] and how they rated their own health with *very healthy* [1], *healthy* [2], *fairly healthy* [3], *unhealthy* [4], and *very unhealthy* [5]*.* A composite score was then calculated to serve as a proximal indicator of actual health.

#### Actual national wealth distribution in Germany and the United Kingdom

Data on wealth for Germany were drawn from a report on wealth distribution by the German Institute for Economic Research (40/2019) [19], and for the UK from the data set “Total Wealth: Wealth in Great Britain” by the National Office for Statistics [20].

#### Calculation

Data were analyzed using SPSS 25.0 (IBM Corp., released 2017). Missing values were excluded pair wise. Stacked bar charts which display all distributions in one figure were used for a visual comparison. For effect sizes, Cohen’s d was calculated for one-sample t-tests, within sample comparisons [24] and between sample comparisons [25].

#### Patient involvement

No patients were involved in the development of the research question, the development of outcome measures, or the design or conduction of this study. No patients were asked to advise on, interpret or write up the results. There are no plans to involve patients in the dissemination of the study results.

## Results

### Estimated, ideal and actual health distributions in Germany and the UK

**Fig. 1:**
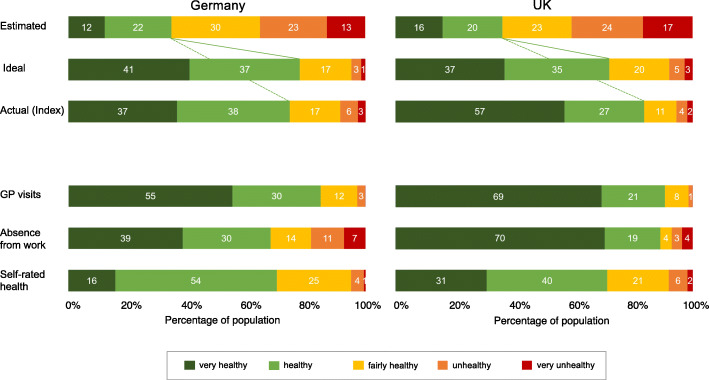
Perceived, ideal, and actual national distribution of health by the five health categories in Germany and the United Kingdom

The estimates for the actual distribution of health and the desired ideal distributions across all five health categories in both countries are presented in Fig. 1, along with the actual distributions for health indicators.

Participants from both Germany and the UK clearly wish for greater health in their societies than they assumed to be the case in their estimates of actual health distributions. While the German and British participants estimated that on average 34% (*SD* = 15.0) and 36% (*SD* = 18.5) of their society were very healthy or healthy, their ideal levels of very good or good health were 78% (*SD* = 21.2) and 72% (*SD* = 20.9), respectively. Thus, the perceived and desired reality diverged by a factor of 2.3 in the German sample (*t* (321) = − 33.44, *p* < .001, *d* = 2.34) and a factor of 2 in the UK sample, (*t* (446) = − 29.70, *p* < .001, *d* = 1.82). Further analyses showed that the two samples did not differ in the estimated distribution of health for their societies (*t* (767) = 1.05, *p* = .293, *d* = 0.08) but did differ slightly, albeit significantly, in the desired distribution (*t* (767) = − 3.96, *p* < .001, *d* = − 0.29).

Compared to the overall index for the actual health distribution, both samples strikingly underestimated the proportion of very good and good health by a factor of 2.2 (estimated 34% vs. actual 75% in the German sample, *t* (321) = − 48.22, *p* < .001, *d* = 2.69) and 2.3 (estimated 36% vs. actual 84% in the UK sample, *t* (446) = − 54.69, *p* < .001, *d* = 2.38). A similar pattern of results emerged when comparing the perceived actual health distribution for each of the three single health indicators. Based on the number of GP visits and the days being absent from work for personal treatment, 85 and 69% in the German sample and 90 and 89% in the UK sample had had less than two GP visits in the last 4 weeks and were absent from work for less than 8 days for personal treatment, and so were considered very healthy or healthy. Compared to the number of GP visits and the days being absent from work for personal treatment, the German and UK samples underestimated the proportion of people with good and very good health by a factor of 2.5 (*t* (321) = − 60.18, *p* < .001, *d* = 3.35 and *t* (446) = − 61.53, *p* < .001, *d* = 2.68) and 2.3 (*t* (321) = − 41.04, *p* < .001, *d* = 2.29 and *t* (446) = − 60.39, *p* < .001, *d* = 2.63), respectively. Comparing the estimated distribution of health to the representative self-rated health revealed that the German (70%: *t* (321) = − 42.23, *p* < .001, *d* = 2.35) and UK (71%: *t* (446) = − 39.87, *p* < .001, *d* = 1.74) samples underestimated the proportion of good and very good health in the national distribution by a factor of 2.0. Country comparisons indicated that these underestimation effects were slightly more pronounced in the UK than in the German sample for the composite health score (*t* (767) = − 7.72, *p* < .001, *d* = 0.56), GP visits (*t* (767) = − 2.94, *p* < .01, *d* = 0.22), and days absent from work (*t* (767) = − 14.90, *p* < .001, *d* = 1.09), but not for self-rated health (*t* (767) = .26, *p* = .799, *d* = 0.02).

Conversely, the difference between the overall index for the actual health distributions and the desired distribution of health in the society was relatively small. In the German sample, the desired proportion of very healthy and healthy people in the society only exceeded the actual proportion by a factor of .04 (78 vs. 75%, (*t* (321) = 2.55, *p* < .05, *d* = − 0.14). In the UK sample, the desired proportion even fell below the actual proportion by a factor of .16 (72 vs. 84%, (*t* (446) = − 12.23, *p* < .001, *d* = 0.58). Positive and negative gaps between the desired and actual health distributions across the UK and German samples resulted in significant country differences (*t* (767) = − 9,82, *p* < .001, *d* = 0.72). Comparing the actual health distribution (based on the three individual health indicators) with the desired distribution showed a similar pattern of results. The desired proportion of very healthy and healthy people differed from the actual proportion (based on the number of GP visits and the days being absent from work for personal treatment) by factors of 0.92 and 1.13 in Germany and 0.80 and 0.81 in the UK, respectively. Compared to the representative self-rated health distribution, both samples equally underestimated the proportion of good and very good health in the national distributions by factors of .11 and .01.

**Fig. 2:**
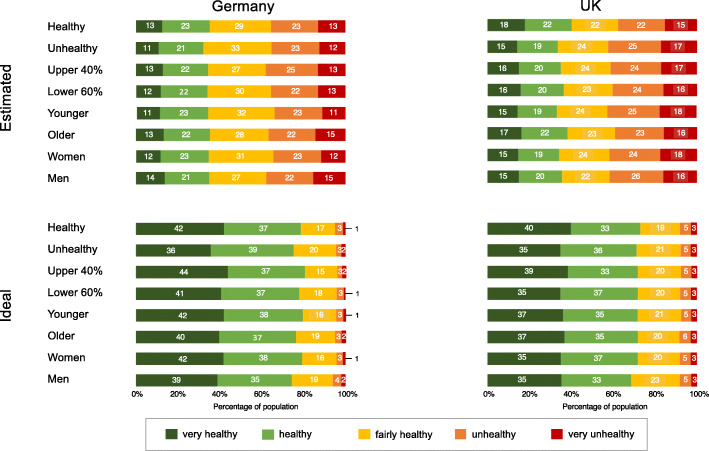
Estimated and ideal national distributions of health by the five health categories for demographic groups in Germany and the United Kingdom. *Note:* The groups were categorized as follows: Healthy (very healthy, healthy) vs. Unhealthy (fairly health, unhealthy, very unhealthy) based on self-rated health (Germany *n* = 266 vs. *n* = 56, UK *n* = 143 vs. *n* = 304); Upper 40% vs. Lower 60% based on self-rated wealth (Germany *n* = 53 vs. *n* = 236, UK *n* = 192 vs. *n* = 249); Younger vs. Older based on median cut on age (Germany ≤25 vs. > 25; *n* = 164 vs. *n* = 158; UK ≤32 vs. > 32; *n* = 233 vs. *n* = 213); Women vs. Men (Germany *n* = 236 vs. *n* = 85; UK *n* = 315 vs. *n* = 130)

In a series of additional analyses, we tested the hypothesis that there is a consensus across demographic groups regarding perceived and ideal health distributions. Figure 2 depicts the estimates for the actual distribution of health and the ideal distribution of health desired by German and British respondents of different genders and ages, and levels of wealth and health. The estimated proportion of very healthy or healthy people in the society ranged between 32 and 36% in the German sample, and between 34 and 40% in the UK sample. In both samples, estimates by the healthy group were the most positive (Germany: 36% and the UK: 40%), in contrast with the unhealthy group, which gave the lowest estimates in both cases (Germany: 32% and the UK: 34%). For desired health, the proportion of very healthy or healthy people in the society ranged between 74 and 81% in the German sample and between 68 and 73% in the UK sample. Estimates from the healthy group (Germany: 79% and the UK: 73%) were slightly more positive than those provided by the unhealthy group (Germany 75% and the UK: 71%). Importantly, the main findings regarding the differences in estimated-desired and estimated-actual health distributions were replicated for each of the four control variables in both the German and UK samples (*t*_*s*_ > − 13.8, *p* < .001).

### Estimated, ideal and actual wealth distributions in Germany and the UK

**Fig. 3:**
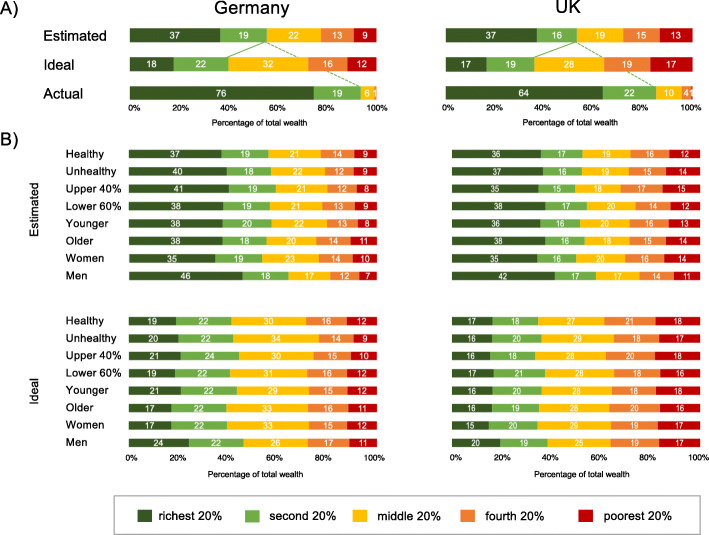
Estimated and ideal national distributions of wealth by the five wealth categories for demographic groups in Germany and the United Kingdom. The groups were categorized as described above

Fig. 3a shows the estimates for the ideal and actual distributions across all five wealth categories for Germany (left panel) and the UK (right panel), along with the actual distributions for the wealth indicators.

Participants from both countries construed ideal wealth distributions that were more equitable than their estimates of the actual distribution of wealth. Both the German and the British participants estimated that about half of the wealth is owned by the upper two quintiles of the population (Germany: 56%; the UK: 53%), but desired a share of 40 and 36%, respectively, resulting in a significant estimated-desired distribution gap in the German (*t* (299) = 9.17, *p* < .001, *d* = − 0.65) and UK samples (*t* (442) = 12.06, *p* < .001, *d* = − 0.67). Further analyses indicated that while the two samples differed in the desired distribution of wealth for their societies (*t* (741) = − 4.58, *p* < .001, *d* = − 0.34), they did not differ in the estimated distribution of wealth (*t* (744) = 1.94, *p* = .053, *d* = 0.15). Compared to the actual wealth distribution in the German and British populations, both samples profoundly underestimated the proportion of wealth owned by the two upper wealth quintiles (actual 95 and 86%) by a factor of 1.7 in the German sample (*t* (302) = − 24.48, *p* < .001, *d* = 1.41) and 1.6 in the UK sample (*t* (442) = 24.01, *p* < .001, *d* = 1.14). As Fig. 3b shows, all the demographic groups shared a consensus in their desire for a more equitable wealth distribution. Notable differences in estimated-desired and estimated-actual wealth distributions were replicated for gender, age, wealth and health groups in both the German and UK samples (*t*_*s*_ > 3.6, *p* ≤ .001).

## Discussion

Overall, the data yielded three main findings. Firstly, there was a broad consensus on ideal and estimated actual health distributions across the German and British samples and across demographic groups: The pronounced gap between ideal and estimated actual health distributions indicates that all groups – even the healthiest participants – would prefer their society to be healthier than their estimation of how healthy it actually was. Secondly, and most noteworthy, the three indicators of the actual health distributions (which were taken from the representative EHIS data sets for Germany and the UK) suggest that the actual distributions are better than the estimated distributions of health, approaching the ideal level of health in the German sample and exceeding expectations in the British sample. Thirdly, these gaps between ideal, estimated, and actual distributions of health are qualitatively different compared to findings in the domain of wealth.

Unlike wealth, in which accumulation in one group of the society is inevitably linked to reduced wealth in other groups, health is not a socially shared commodity and entitlements to health can be construed as an aspect of citizenship [26]. However, rather than aiming for perfect health for the whole population, the yardstick for the ideal distribution of very good or good health is set at 78 and 72% of the German and UK populations, respectively. People seem willing to accept that, to some degree, detrimental conditions lead to a situation in which around a quarter of the population have a poor or modest health status. Beyond biological predispositions, there is strong evidence that health is dependent on multiple factors ranging from individual decisions and habits to structural factors including income and social position, which operate at various levels such as poor nutrition, health behaviors, working and life conditions, access to health systems, and public policy [27–29, 1]. More than the fact of existing health differences in the population as such, views on the causes of ill-health might influence acceptance and perceived fairness of health differences. In this, health inequalities attributed to economic or environmental circumstances as compared to individual predispositions or decisions seem deemed as particularly unfair and unjust [26, 30, 31].

The broad consensus that was observed across both samples and all demographic groups for ideal health distributions suggests that German and British participants may share a “normative” standard for the distribution of health, despite having different health care systems and disagreeing about measures and policies that affect health distribution. “Normative” in this context is merely a descriptive term (see 11) which indicates that participants from different countries and demographic backgrounds agree on the ideal distribution of health in their societies. A society might have the level of health that participants construe as ideal but this might still not fully realize its actual potential, nor be optimal from a public health or economic perspective.

The consensus that was observed for ideal health distributions also extended to the estimated actual distribution of health in the society. Woman and men, younger and older, healthier and less healthy, rich and less rich groups all provided similar estimated distributions of their societies’ actual health, with 34% of the German and 36% of the UK populations rated as being very healthy or healthy. The data indicates a profound gap between ideal and estimated actual distributions of health, which differed by a factor of more than 2 regarding the proportion of people who should be and were estimated to be in good or very good health. The gap between ideal and estimated actual health distributions may serve as a proxy for evaluating the performance of public health care. This may in turn affect the framing of debates on health care systems, with the “felt” gap between the nation’s ideal and estimated actual health potentially leading to a focus on negative developments, funding issues and challenges, rather than a comprehensive picture including positive developments and opportunities.

While people seemingly misperceive the distribution of both health and wealth, the direction of misperception was in opposite direction. Previous studies comparing estimated actual and ideal wealth distributions to actual wealth distributions have produced consistent results across different samples and nations [32, 10]. The present data replicate these findings, since the participants’ underestimations of the actual amount of wealth inequality reveal an even larger gap between estimated-actual than ideal-actual wealth distributions. These positive views on actual wealth distribution contrast with rather negative ones on health. While participants positively misperceived the current level of wealth inequality, suggesting that they are unaware of the actual wealth gap in their societies, they negatively misperceived the current level of health inequality. As the assessment method for perceived and ideal health and wealth distributions were comparable, the observed gaps between distributions does not seem to be caused by a general perception bias, rather inequality perceptions were modulated by the respective topic. One might speculate that health perceptions are more pessimistic as various influencing factors such as information provided through the media, social networks, health care provides etc. is skewed towards “bad news” and diseases. Moreover, assessing perceived and ideal health distributions for different wealth groups in the population probably will result in a differential pattern of results (e.g., Marmot Review [1]). Specifically, people might underestimate the health in the population but overestimate the health in vulnerable groups.

### Strengths and weaknesses of the study

There are some caveats which need to be considered for the present data. Regarding actual health distributions, our composite index includes GP visits and absence from work, which may provide an overly optimistic picture of the actual distribution of health. For instance, one can be sick and nonetheless go to work or decide not to visit a doctor. We readily acknowledge that other measures of health may possibly reveal different and more negative views on a nation’s health. However, there is currently neither a gold standard of a composite nor a single measure of individual health indicators available which is consensually agreed. What we find most surprising is that the data on the distribution of self-reported health collected from representative German and UK samples within the framework of EHIS are rather similar to the ideal health distribution and sharply more positive than the estimated health distribution within countries. Furthermore, the German and UK samples are not demographically representative of the German or British populations. Compared to the overall population, the German and British samples were younger, included more female participants, and were better educated. However, while not representative, the robustness of the findings across several relevant demographic groups, i.e. age, gender, health status, and similarity of findings across the German and UK samples, despite profound differences in health systems, is reassuring and invites an examination of representative samples across a wider range of nations.

### Implications of the study

(Mis) perceptions of health and wealth may substantially affect policy endorsement. For instance, the Marmot Review [1] states that “… health follows a social gradient. Everyone below the top has greater risk of worse health than those at the top” (page 7.), and suggests the setting of strategic goals to reduce health inequality. It is presumed that perceptions of the steepness of the social gradient, and how great the challenge to achieve a higher level of health actually is, will affect societal agreement with health policy campaigns. Furthermore, the distributions of the actual and ideal health data were rather similar, leading to the surprising hypothesis that the status of their nation’s health is actually better than the participants from Germany and the UK perceived. While research has yet to demonstrate a strong relationship between the desired distribution of health and the endorsement of ensuing policies [10, 11], awareness that ideal and actual health distribution are more similar would send a positive message, possibly facilitating a mindset of opportunity to further increase the level of health in the population. One might speculate that the health related information provided by for example the media and social networks is more skewed towards “bad news” as compared to wealth related information which might have contributed to the observed discrepancy between the two topics.

Given that most people are not experts on public health, they presumably report reflective interpretations and beliefs about the perceived actual situation, which in turn presumably build upon intuitive processes and different sources [9]. One line of reasoning reflects social comparison processes. Specifically, people often believe that they are healthier than their peers, indicating unrealistic optimism ([33], for an overview see [34]). The present data support this notion, since self-reported health distributions from the representative EHIS data sets for Germany and the UK were profoundly more positive than the estimated actual health distributions. Another possibility is that negative health perceptions may reflect sampling rates and a greater exposure to negative health information in the public and media. A tendency towards negative perceptions has been observed for a range of topics of public interest such as violence [35] or political news [36]. Similarly, the media contains many advertisements promoting the health benefits of various products and campaigns requesting changes in life-style and health behaviors, and political debates about the increasing costs of health systems, often accompanied by concerns about inefficacy, may contribute to the impression of a comparably unhealthy rather than healthy nation. Finally, negative perceptions of the actual distribution of health may derive from an increasingly high standard of health. Research has shown that changes in concepts can occur when extreme cases are less frequently encountered [37]. Accordingly, when people experience serious health threats less frequently, a shift in concept may occur in which less severe health threats are now seen as riskier. Thus, a prevalence induced concept shift of health may also lead to a pessimistic view on the nation’s health. It is worth noting that considering health beliefs as reflective and situation-bound interpretations of intuitions entails the notion that such beliefs are flexible and depend on stimulus input, i.e. having a sensible ratio between positive and negative information and processing goals, i.e. promotion vs. prevention. Insights into the fundamental processing characteristics of beliefs can be used to improve the discourse of public health professionals and, for instance, address overly pessimistic views on the national health distribution. However, making people aware of the gap can potentially lead to changes in their perceptions without necessarily changing their pessimistic outlook. Considering results from other fields, i.e., examining biases such as unrealistic optimism in health risk perceptions [38, 39, 32], one may assume that people may adapt their pessimistic perception of the current level of health in the population while simultaneously also changing their ideal health distribution, which may preserve the observed gap between perceived and actual health distributions.

### Outlook and future research

While the present study provides an important first step in examining perceptions of health inequality and their accuracy, it also raises new issues. Future research should therefore investigate the reasons for negative misperceptions of health, as well as the implications these misperceptions might have on trust in public health services, social and economic policies, and policymakers. Moreover, building on the present results, future studies should try to foster the integration of the subjective perspective into research on health inequalities, as well as investigating environmental and structural aspects and their complex associations with perceived, ideal and actual health. Monitoring and providing the public with assessable, transparent and reliable information about objective health and wealth indicators in the population, for example by using data visualizations, seem therefore to be key for facilitating accurate perceptions and public trust. This in turn, may also enable discussions about the causes of and opportunities for improving health and health equity.

## Conclusion

People’s beliefs about ideal and actual health and wealth distributions provide general information of how these two essential domains are perceived and desired to be. The present findings show that people have an overly pessimistic view on the distribution of health in their societies, which contrasts with optimistic misperceptions of the distribution of wealth. While the participants believed that there is a profound negative gap between the actual and desired level of health in their societies, ideal health distributions approached or even exceeded actual health distributions. Conversely, the current level of wealth inequality was profoundly underestimated. Most importantly, from a public health and policy perspective, a high level of consensus emerged across demographic groups: both healthy and wealthy participants misperceived health and wealth distributions. Hence, addressing misperceptions and informing the public about the ideal-estimated-actual gap may help to prevent overly negative views on the nation’s health, which may be attributed to the health system and undermine trust and confidence.

## Supplementary Information

**Additional file 1.**

## Data Availability

Macro data for the EHIS 2 can be downloaded free of charge on the Eurostat website, access to the micro data can be requested from Eurostat via a research contract. Deidentified survey data may be made available on request. Proposals should be directed to britta.renner@uni-konstanz.de. To gain access, data requestors will need to sign a data access agreement.
