# Supplementary material for: Pessimistic health and optimistic wealth distributions perceptions in Germany and the UK: evidence from an online-survey
Source: BMC Public Health. 2021 Jul 3;21:1306. doi: 10.1186/s12889-021-11355-x (PMC8254428; doi:10.1186/s12889-021-11355-x)
Supplement: Supplementary file 1 — Additional file 1. [file 12889_2021_11355_MOESM1_ESM.pdf]

Dear participant,

Fitness, health and lifestyle play important roles in our everyday lives, and we are interested in your opinion on these topics for this scientific study.
Since the research results will be more reliable and precise if more people participate in the survey, we would be very grateful if you would spend 10-15 minutes completing this questionnaire.

The questionnaire is open to everyone from within the general population in the UK, regardless of age, and without any inclusion criteria or specific target group. After completing the questionnaire you can choose to be included in a raffle in which you can win one of 10 Amazon vouchers, each worth £50.

Please read the following questions carefully, and then tick the answers which you think best apply to you, personally, or which most accurately reflect what you believe. There are no “right” or “wrong” answers; the only thing that matters is your personal assessment. If you find it difficult to decide your answer to a question, just give whichever answer you think applies the most.

All your information will of course be processed anonymously, kept confidential, and only used for scientific purposes. It is impossible to relate any answers to a specific participant.
Your participation in this study is entirely voluntary and you can withdraw at any time, without giving a reason or prejudicing your rights, and without any negative effects or sanctions. However, only people who complete the questionnaire fully will be included in the raffle for Amazon vouchers.

If you have any further questions about this study, please do not hesitate to contact us.
Thank you very much for your interest and your participation!

I have read and understood the information provided and would like to participate in this study.

- ◯ yes
- ◯ no

I am...

- ◯ female
- ◯ male
- ◯ other

Your year of birth:

☐

How healthy are you?

- ◯ very healthy
- ◯ healthy
- ◯ fairly healthy
- ◯ unhealthy
- ◯ very unhealthy

How healthy is the UK?

Countries differ in respect to how healthy their population is. For example, people living on the Japanese island of Okinawa are predominantly very healthy, and have one of the highest life expectancies in the world.
We would now like you to assess the population of the UK with respect to their health and fitness.

Health in the UK: What proportion of the population has very good or very bad health?

People living in the UK can be expected to fit into one of the following five categories: very healthy, healthy, fairly healthy, unhealthy, and very unhealthy. Please indicate below what percentage of the UK's population you think fits into each of these fivve categories.

Example:
If you think that most of the population is "very healthy", and that people are evenly spread amongst the other categories, then you might assign the following percentages: very healthy 60%, and all other categories 10% each.

Please indicate which percentage of the UK population you feel fits into the following levels.

The total percent that you assign for the five levels should add up to 100%.

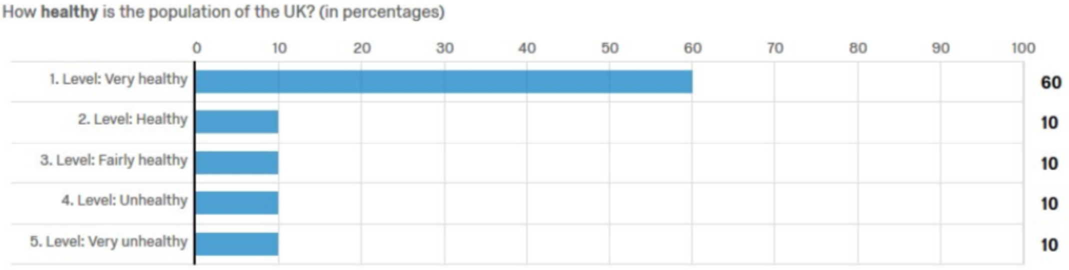

How <u>healthy</u> is the population of the UK?
Please indicate percentages.

The total percent that you assign for the five levels should add up to <u>100%</u> .

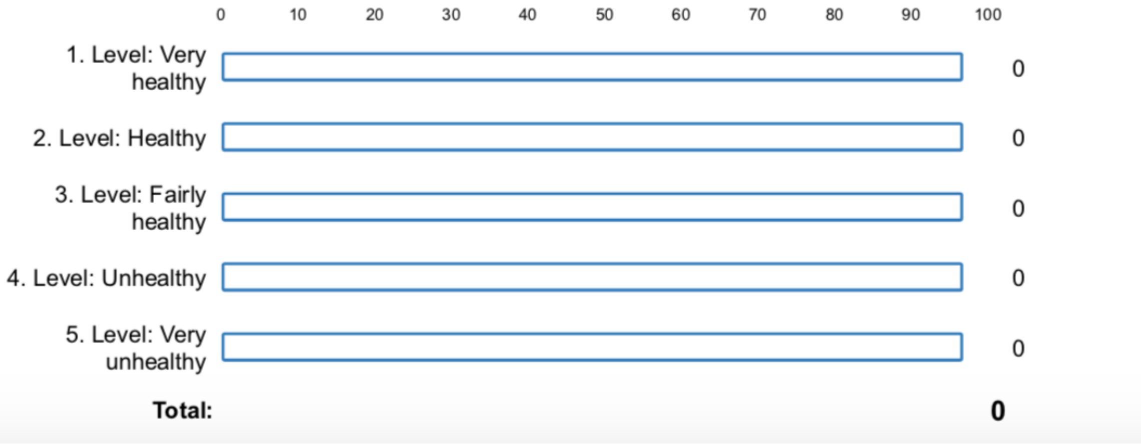

In your opinion, how healthy should the population of the UK ideally be?
Please indicate which percentage you feel should fit into the following levels.

The total percent that you assign for the five levels should add up to 100% .

	0	10	20	30	40	50	60	70	80	90	100
1. Level: Very healthy											
2. Level: Healthy											
3. Level: Fairly healthy											
4. Level: Unhealthy											
5. Level: Very unhealthy											
**Total:**											

How is wealth distributed in the UK?

The wealth of a country is distributed amongst its population.

Wealth, also known as net worth, is defined as the **total value of everything someone owns minus any debt(s) that they owe**. A person's net worth includes their bank account savings plus the value of other things such as property, stocks, bonds, pension, art, collections, etc., minus the value of things like loans and mortgages.

For this question, we divide the UK's population into five groups, each consisting of 20% of the households.

Please indicate what percentage of the UK's <u>total wealth</u> you feel is owned by each group.

The total percent you assign for the five levels should add up to <u>100%</u>.

	0	10	20	30	40	50	60	70	80	90	100
1. Group: Poorest 20%											
2. Group: Second 20%											
3. Group: Middle 20%											
4. Group: Fourth 20%											
5. Group: Richest 20%											
**Total:**											

In your opinion, how <u>should</u> the total wealth be distributed amongst these five groups? Please indicate percentages.

The total percent you assign for the five levels should add up to <u>100%</u>.

	0	10	20	30	40	50	60	70	80	90	100
1. Group: Poorest 20%											
2. Group: Second 20%											
3. Group: Middle 20%											
4. Group: Fourth 20%											
5. Group: Richest 20%											
**Total:**											

Based on my <u>current financial situation</u>, I would consider myself as belonging to the following group:

- ◯ Poorest 20% of the population
- ◯ Second 20% of the population
- ◯ Middle 20% of the population
- ◯ Fourth 20% of the population
- ◯ Richest 20% of the population
